# Treatment of stroke related refractory brain edema using mixed vasopressin antagonism: a case report and review of the literature

**DOI:** 10.1186/s12883-014-0213-0

**Published:** 2014-11-18

**Authors:** Vishnumurthy Shushrutha Hedna, Sharathchandra Bidari, David Gubernick, Saeed Ansari, Irawan Satriotomo, Asif A Khan, Adnan I Qureshi

**Affiliations:** Departments of Neurology, College of Medicine, University of Florida, Room L3-100, McKnight Brain Institute, 1149 Newell Drive, Gainesville, FL 32611 USA; Radiology, University of Florida, Gainesville, FL USA; Surgery, University of Florida, Gainesville, FL USA; Department of Neurology, Central Care Health, St. Cloud, MN USA

**Keywords:** Cerebral edema, Stroke, Vasopressin, Mixed vasopressin antagonism, Conivaptan

## Abstract

**Background:**

Elevated intracranial pressure from cerebral edema is the major cause of early mortality in acute stroke. Current treatment strategies to limit cerebral edema are not particularly effective. Some novel anti-edema measures have shown promising early findings in experimental stroke models. Vasopressin antagonism in stroke is one such target which has shown some encouraging preliminary results. The aim of this report is to highlight the potential use of vasopressin antagonism to limit cerebral edema in patients after acute stroke.

**Case presentation:**

A 57-year-old Caucasian man with new onset diplopia was diagnosed with vertebral artery aneurysm extending into the basilar circulation. He underwent successful elective vertebral artery angioplasty and coiling of the aneurysm. In the immediate post-operative period there was a decline in his neurological status and brain imaging revealed new midbrain and thalamic hemorrhage with surrounding significant brain edema. Treatment with conventional anti-edema therapy was initiated with no significant clinical response after which conivaptan; a mixed vasopressin antagonist was started. Clinical and radiological evaluation following drug administration showed rapid clinical improvement without identification of significant adverse effects.

**Conclusions:**

The authors have successfully demonstrated the safety and efficacy of using mixed vasopressin antagonist in treatment of stroke related brain edema, thereby showing its promise as an alternative anti-edema agent. Preliminary findings from this study suggest mixed vasopressin antagonism may have significant utility in the management of cerebral edema arising from cerebrovascular accident. Larger prospective studies are warranted to explore the role of conivaptan in the treatment of brain edema and neuroprotection.

## Background

Stroke is the fourth leading cause of death in United States [1]. Brain edema and herniation are implicated in the majority of these cases [2]. No effective agents exist that have altered the management of brain edema to the satisfaction of clinicians involved in stroke care. Decompressive hemicraniectomy has reduced mortality in malignant middle cerebral artery (MCA) stroke, but only when used in younger populations within 48 hours of symptom onset [3]. In the neurocritical care setting, mannitol and hypertonic saline are used extensively for managing brain edema due to the lack of more effective options, rather than its therapeutic superiority [4]. Significant controversy about the advantages and disadvantages of these agents in long term patient outcomes following brain edema further complicate the clinical picture [5]. Hence there is a great need for alternative agents to rapidly decrease increased intracranial pressure as a result of stroke-related brain edema, thereby reducing brain herniation and its subsequent morbidity and mortality.

Arginine-vasopressin (AVP), a potent endogenous hormone responsible for regulating plasma osmolality and volume, has demonstrated a role in the pathophysiological mechanisms in stroke [6,7]. Evidence of AVP’s significant role in cerebral edema has made it a promising drug target in the management of this condition [8]. Chang *et al*. found time-dependent increases in serum AVP levels after brain injury as well as attenuation of AVP levels following administration of 7.5% hypertonic saline in an experimental stroke model [7]. These studies also found that osmotherapy is effective in reducing intracranial pressure (ICP) through a common AVP-mediated pathway.

The mechanism of action of AVP is mainly mediated by 2 receptor subtypes: V1a and V2 which are expressed in the brain, pituitary gland, myocardium, vasculature and kidneys. Experimental models have demonstrated the utility of V1a and V2 AVP receptor antagonism in attenuation of ischemia related cerebral edema and infarct volume by aquaporin (AQP) 4 expression modulation. Even though appealing, the evidence for clinical utility of vasopressin antagonism in stroke related brain edema is sparse. Conivaptan is a mixed vasopressin receptor (V1a and V2) antagonist that belongs to the group of non-peptide vasopressin antagonists referred to as “Vaptans” [9]. This class has been approved by Food and Drug Administration (FDA) for use in hypervolemic/euvolemic hyponatremia [10].

We report a case of a disabling stroke after an endovascular procedure who received conivaptan as last resort to reduce his brain edema. This patient’s clinical course and radiological findings were serially monitored and recorded. Adverse events and safety data from this medication were also monitored and documented.

## Case presentation

A 57-year-old Caucasian male with residual right-sided hemiparesis from a cerebrovascular event, 1 month prior to this admission, presented with sudden onset of vision changes. He complained his vision was “upside down” with associated headache, nausea and vomiting. His past medical history included pacemaker implantation, and multiple sclerosis in remission. On neurological exam, his National Institutes of Health Stroke Scale (NIHSS) was 9 when including his previous residual neurological deficits. Higher cognitive function was mostly intact except for dysarthria, and diplopia on horizontal gaze with right internuclear opthalmoplegia. His old deficits from recent stroke included partial right facial palsy with right hemiparesis (motor system examinations using the Medical Research Council (MRC) grade: 2/5 in right upper and lower extremity), hemi body numbness and intact cerebellar function. Computed tomography (CT) scan of the head without contrast revealed left precentral gyrus hypodensity extending to the hand area, most consistent with an evolving late subacute infarct corresponding to the patient's right upper extremity weakness. Most prominent was the finding of a partially calcified 16 mm fusiform aneurysm of the left vertebral artery (VA) with extension to the basilar junction and beyond. On day 1 of admission, he was complaining of severe intractable headaches and depression. On day 6, he had a sudden decline in his neurological exam wherein he developed complete facial weakness, became somnolent, and suffered a 25 minutes generalized tonic clonic seizure (which was treated with a standard status epilepticus treatment regimen). Repeat head CT demonstrated new hypodensity in the mesial aspect of the left midbrain compared to the admission study. This was most consistent with a subacute infarct in the basilar perforator territory which explained the patient's double vision.

**Figure 1:**
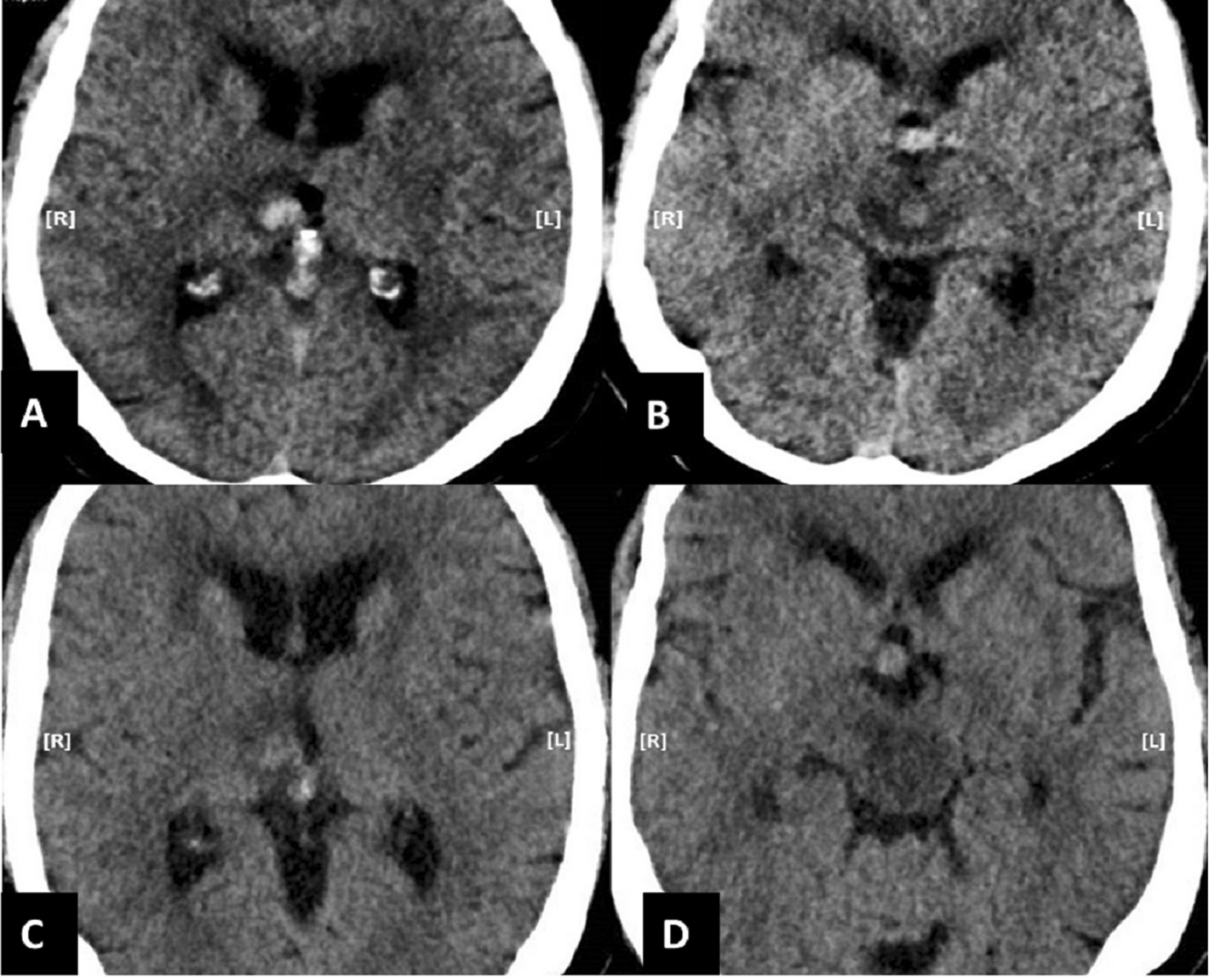
**Imaging before conivaptan administration.** The immediate post-procedure (intra-arterial stent insertion) CT head (without contrast) demonstrate acute hemorrhage in the right mesial thalamus **(A)** with surrounding mild vasogenic edema and hypodensity on both sides of mid brain **(B)**. The CT head (without contrast) before conivaptan administration reveals slight decrease in the density of acute hemorrhage in the right mesial thalamus, but with worsening surrounding vasogenic edema **(C)**. The hypodensity in the mid brain has also worsened without any normal density tissue visible, especially adjacent to the quadrigeminal cistern **(D)**.

Elective right vertebral artery angioplasty with aneurysm stenting of the vertebral artery was performed on day 13. Approximately 2 hours after the procedure his left pupil became fixed and dilated. He developed dense hemiplegia on his left side. Stat head CT demonstrated interval development of acute hemorrhage in the mesial aspect of right thalamus measuring 11 × 9 mm in maximal axial dimensions. There was also hemorrhage seen in the central part of upper midbrain measuring 8 × 8 mm. A significant vasogenic edema surrounding the hematoma that extended up to the lower midbrain was seen (Figure 1A, B).

**Figure 2:**
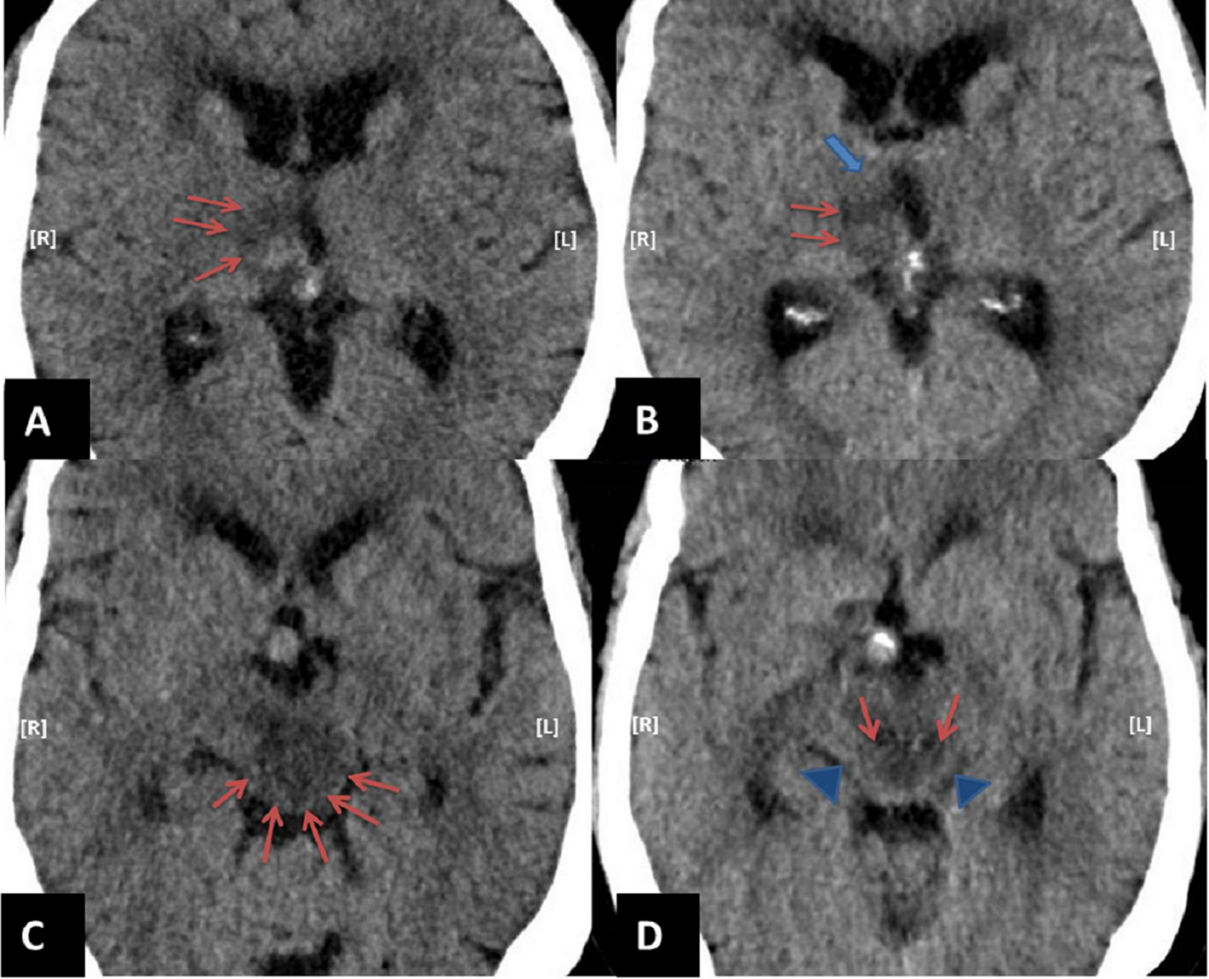
**Imaging after conivaptan administration.** Comparative images at the level of thalamus pre **(A)** and post **(B)** conivaptan administration shows significant reduction in vasogenic edema (red arrows) with normal density appearing tissue seen in the ventral aspect of right thalamus following conivaptan administration (thick blue arrow). Moreover, comparative images at the level of mid brain pre **(C)** and post **(D)** conivaptan administration clearly demonstrate significantly reduced hypodensity (red arrows), especially adjacent to the quadrigeminal cistern. The normal density appearing tissue emerges following conivaptan administration (blue arrow heads).

He was started on hypertonic saline with a target serum osmolality of 300–320 mOsm/kg and serum sodium goal of 150–160 mEq/L (post-operative sodium was 140 mEq/L). On post-operative day 1, there was no change in his neurological exam as the patient remained unresponsive with left fixed, dilated pupil and no movement on his left upper and lower extremity. There was interval increase in the brain stem edema in comparison to brain imaging from the previous day (Figure 1C, D). Due to the lack of response from hypertonic saline, he was started on intravenous conivaptan. Conivaptan was administered as per the drug administration guidelines approved by FDA with 20 mg infused over 30 minutes as a loading dose, followed by a continuous infusion of 20 mg over 24 hours (0.83 mg/hour) planned for 2 days. On post-operative day 2, after 8 hours of receiving the loading dose of conivaptan, patient started responding to commands and was seen moving his left upper and lower extremities. His sodium was 145 mEq/L. In the next 2 days he was showing significant progress on neurological exam, even demonstrating some strength in his left upper extremity. Repeat head CT on day 18 (after 2 days of conivaptan administration) demonstrated expected hematoma density changes both within the mesial right thalamus and in the central midbrain, but the size of the hematoma was stable compared to prior examination. However, the perilesional edema had significantly decreased, most apparent in the upper and mid portion of the midbrain. The comparison of the imaging at the level of both thalamus (Figure 2A, B) and midbrain (Figure 2C, D) showed noticeable resolution of brain edema and restoration of some normal tissue after conivaptan administration. Despite making significant neurological improvements and tolerating extubation our patient subsequently developed respiratory failure presumably due to aspiration pneumonia and his family opted to shift to palliative care only.

## Discussion

Conivaptan is a mixed vasopressin antagonist, currently approved for euvolemic or hypervolemic hyponatremia in heart failure, which has shown tremendous promise in both experimental and clinical brain injury scenarios. It acts via both V1a and V2 receptors making it the only FDA approved mixed vasopressin antagonist [9]. There is strong literature evidence to show V1a antagonism is useful in limiting vasopressin mediated brain damage after inducing stroke in animal models. The V1a antagonism was helpful in reducing brain edema via down-regulation of AQP-4 in these experiments [11]. Since the V2 receptor mediates AVP expression via positive feedback, V2 antagonism may, in theory, be advantageous when formulating novel ligands for neuroprotection. In the neurocritical care literature, conivaptan has been used safely to reduce intracranial pressure, increase serum sodium to desired goal, and to augment cerebral perfusion pressure [12,13]. There is anecdotal evidence for its use in neurosurgical and cardiac intensive care unit (ICU) settings where it show promise in the treatment of hyponatremia secondary to traumatic brain injury and other conditions [14,15]. The most common side effects described for the short-term use of conivaptan include infusion site phlebitis, headaches, increased thirst, pyrexia and constipation. Orthostatic hypotension, hypokalemia, rebound hyponatremia, atrial dysrhythmias, and sepsis are other serious adverse effects. Contraindications for using conivaptan are hypovolemic hyponatremia and simultaneous use of CYP3A4 inhibitors [13].

During stroke, AVP is released from the posterior pituitary in response to histamine activation of magnocellular neurons in the hypothalamus AVP then interacts with its receptors V1a and V2 to mediate processes which lead to further cerebral edema. Antagonism of these receptors is described below:

### Role of V1a receptors in brain

Vasopressin receptor V1a is involved in the pathogenesis of secondary brain damage following focal cerebral ischemia. The V1a receptor antagonism causes platelet inhibition, AQP-4 channel modulation, reduced infarct size and vasodilation. Also, V1a antagonists have been shown to prevent the development of ischemia-induced cerebral edema, thereby suggesting the importance of the V1a receptor interaction in water regulation in brain cells [16-18]. Further, vasopressin V1a receptor antagonism has largely been shown to improve outcomes after experimental stroke. A V1a antagonist SR49059, has been demonstrated to decrease rates of brain water accumulation at varying time points in intracerebral hemorrhage (ICH) [19,20]. This agent also reduced AQP-4 expression and led to decreased cerebral edema after ICH. The aforementioned antagonist was found to reduce cytotoxic brain edema via AQP-4 attenuation in an experimental model of middle cerebral artery occlusion (MCAO) [11]. Hence, it appears that AQP-4 channels, which play a key role in development of brain edema, may be modulated by V1a antagonism [9]. Other V1a antagonists have also shown similar effects in reducing brain water content after cold-induced edema in addition to dose-dependent decreases in the permeability of the blood–brain barrier [8]. The V1a antagonism mediated neuroprotection is postulated to occur via dampening of cerebrovasodilation, caused by ATP-sensitive and calcium-sensitive potassium channels, potentially leading to decreased reperfusion injury and vasogenic edema [21]. Vakili *et al*. successfully demonstrated V1a antagonism resulted in a dose-dependent attenuation of infarct volume and blood–brain barrier disruption and more importantly a decrease in brain edema [8].

### Role of V2 receptors in brain

The V2 receptor expression in the central nervous system is limited and the extent of its contribution in brain edema is thus controversial [22]. Some studies demonstrate V2 receptor antagonism may act by decreasing AVP release through a negative feedback mechanism [23,24]. Recently, a few studies have demonstrated that the V2 receptor antagonist, OPC-31260, may be effective in the early treatment of cytotoxic edema and brain injury [18,25]. Administration of OPC-31260 ameliorated cerebral neurological deficit in transgenic mice overexpressing endothelin-1 in astrocytes (GET-1) after water intoxication. Treatment with OPC-31260 also significantly decreased water accumulation and down-regulated AQP-4 expression levels in GET-1 mice after water intoxication. In fact, AQP-4 levels were shown to almost be completely normal when comparing AQP-4 staining intensity in GET-1 mice with and without water intoxication. It was also shown that administration of OPC-31260 at doses of 10 to 30 mg/kg led to a dose-dependent inhibition of subarachnoid hemorrhage-induced cerebral edema formation, accompanied by an increase in urinary volume and decrease in urine osmolality without a significant alteration of urine electrolytes [26]. The V2 receptor antagonism also helped diminish water accumulation, decreased glial fibrillary acidic protein (GFAP) in astrocytes, and most importantly led to a renal tubule-selective diuretic effect called aquaresis (electrolyte sparing diuresis), which may have additional benefits in the reduction of cerebral edema [12].

To further explore the exciting promise of Conivaptan’s use in stroke and brain edema, we have developed an experimental murine stroke model [27]. Early results from this study have been promising. We have demonstrated that conivaptan ameliorated brain swelling at two early time points (12 h and 24 h) by more than 50% in comparison to control in MCAO. At 12 h, ipsilateral average hemispheric edema (HE%) in the conivaptan-treated group (n = 16) was 6.64 ± 1.62% versus 16.55 ± 1.76% in controls (n = 16, p = 0.0003). This was reproduced at 24 h, where HE% in the conivaptan-treated group (n = 12) was 6.81 ± 1.33% in comparison to 13.93 ± 1.57% in the control group (n = 12, p = 0.002). Our studies are underway to answer some questions about its mechanism of action in attenuation of brain edema by concentrating specifically on aquaporin channels and its ligands.

## Conclusions

Brain edema contributes significantly to morbidity and death associated with stroke. However current option treatments are limited to hyperosmolar agents and surgical decompression. In this case report we describe the potential of the mixed vasopressin antagonist, conivaptan, in the acute treatment of brain edema. To our knowledge, this is the first case report in the literature describing the benefits of conivaptan use in clinical brain edema. Although this report is very encouraging, additional randomized clinical studies and caution is advised regarding its use until the result of more definitive trials investigating its safety and efficacy are known. We hope that this report highlights the inadequacy of standard treatment of cerebral edema and generates future studies which explore novel therapeutic agents, including larger studies to fully characterize the potential role of mixed vasopressin antagonism.

## Consent

Written informed consent was obtained from the patient's next of kin for publication of this Case report and any accompanying images. A copy of the written consent is available for review by the Editor of this journal.

## Abbreviations

MCA: Middle cerebral artery
MCAO: Middle cerebral artery occlusion
ICP: Intracranial pressures
AVP: Arginine-vasopressin
AQP: Aquaporin
FDA: Food and Drug Administration
NIHSS: National Institutes of Health Stroke Scale
MRC: Medical Research Council
CT: Computed tomography
VA: Vertebral artery
ICH: Intracerebral hemorrhage
HE: Hemispheric edema
ICU: Intensive care unit
